# Abnormal Thyroid Hormone Status Differentially Affects Bone Mass Accrual and Bone Strength in C3H/HeJ Mice: A Mouse Model of Type I Deiodinase Deficiency

**DOI:** 10.3389/fendo.2019.00300

**Published:** 2019-05-15

**Authors:** Clarissa R. Zaitune, Tatiana L. Fonseca, Luciane P. Capelo, Fatima R. Freitas, Eduardo H. Beber, José M. Dora, Charles C. Wang, Manuela Miranda-Rodrigues, Keico O. Nonaka, Ana L. Maia, Cecilia H. A. Gouveia

**Affiliations:** ^1^Department of Anatomy, Institute of Biomedical Sciences, University of São Paulo, São Paulo, Brazil; ^2^Institute of Healthy Sciences, Paulista University, São Paulo, Brazil; ^3^Section of Adult and Pediatric Endocrinology, Diabetes and Metabolism, Department of Medicine, University of Chicago, Chigago, IL, United States; ^4^Institute of Science and Technology, Federal University of São Paulo, São Paulo, Brazil; ^5^Heart Institute (InCor) of Medical School Hospital, University of São Paulo, São Paulo, Brazil; ^6^Department of Morphology, Health Sciences Center, Federal University of Espirito Santo, Vitoria, Brazil; ^7^Endocrine Division, Hospital de Clinicas de Porto Alegre, Federal University of Rio Grande do Sul, Porto Alegre, Brazil; ^8^São Carlos Institute of Physics, University of São Paulo, São Carlos, Brazil; ^9^Department of Physiological Sciences, Federal University of São Carlos, São Carlos, Brazil; ^10^Department of Physiology and Pharmacology, University of Western Ontario, London, ON, Canada

**Keywords:** thyroid hormone, iodothyronine deiodinases, bone mass accrual, bone growth, C3H/HeJ and C57BL/6J

## Abstract

C3H/HeJ (C3H) mice are deficient of type I deiodinase (D1), an enzyme that activates thyroid hormone (TH), converting thyroxine (T4) to triiodothyronine (T3). Nevertheless, C3H mice present normal serum T3 and a gross euthyroid phenotype. To investigate if a global D1 deficiency interferes in the TH effects on bone, we compared bone growth, bone mass accrual and bone strength of C3H and C57BL/6J (B6) mice under abnormal TH status. Four-week-old female mice of both strains were grouped as Euthyroid, Hypothyroid (pharmacologically-induced), 1xT4 and 10xT4 (hypothyroid animals receiving 1- or 10-fold the physiological dose of T4 /day/16 weeks). Hypothyroidism and TH excess similarly impaired body weight (BW) gain and body growth in both mice strains. In contrast, whereas hypothyroidism only slightly impaired bone mineral density (BMD) accrual in B6 mice, it severely impaired BMD accrual in C3H mice. No differences were observed in serum and bone concentrations of T3 between hypothyroid animals of both strains. Interestingly, treatment with 10xT4 was less deleterious to BMD accrual in C3H than in B6 mice and resulted in less elevated T3 serum levels in B6 than in C3H mice, which is probably explained by the lower D1 activity in C3H mice. In addition, hypothyroidism decreased bone strength only in C3H but not in B6 mice, while TH excess decreased this parameter in both strains. These findings indicate that D1 deficiency contributes to the TH excess-induced differences in bone mass accrual in C3H vs. B6 mice and suggest that deiodinase-unrelated genetic factors might account for the different skeleton responses to hypothyroidism between strains.

## Introduction

Thyroid hormone (TH) is essential to bone development and metabolism. Hypothyroidism leads to a delay in skeletal maturation and epiphyseal disgenesis, resulting in reduced longitudinal growth and skeletal abnormalities (1). On the other hand, TH excess may result in accelerated skeletal maturation with premature closure of the epiphyseal growth plates and a consequent decrease in the growth of the limbs (1, 2). In hyperthyroidism, there is also an increase in bone metabolism, where the osteoclastic activity predominates favoring a negative balance of calcium and bone loss (3). Of note, overt thyrotoxicosis is a well-recognized cause of secondary osteoporosis (4–6). In contrast, hypothyroidism leads to reduced bone metabolism with no change or a slight increase in bone mass (7). However, animal studies show that TH deficiency greatly impairs bone mass acquisition during development due to an important decrease in bone formation (8, 9). Therefore, TH exerts critical anabolic actions in the bone tissue during skeletal development.

There is a general consensus that most TH actions are mediated by its nuclear receptors (TRs), which were shown to be expressed in skeletal cells (10–12). To exert its nuclear activity, thyroxine (T4), which is the major secretory product of the thyroid gland, needs to be converted to triiodothyronine (T3) by the action of type I and type II iodothyronine selenodeiodinases (D1 and D2, respectively). Since D1 is a plasma membrane protein, it offers ready access of circulating T4 to the enzyme and facilitates the entry of the D1-generated T3 into the plasma (13). Therefore, D1 contributes to the circulating T3 concentration, but has minimal contribution to the intranuclear T3 content (13–15). On the other hand, T3 produced via D2, an endoplasmic reticulum membrane-resident protein (14), easily reaches the cell nucleus. Therefore, D2 significantly contributes to the local (cell-tissue-organ) T3 content, besides contributing to the serum levels of T3 (13). D1 activity is restricted to the thyroid gland, liver, and kidney, (14), whereas D2 is highly expressed in the brain and in many other tissues (16, 17) including bone and cartilage (18, 19). Given this widespread D2 expression throughout the body, D2 activity is thought to be the major contributor for T3 serum production with a minor contribution from D1 (20). D1 is activated by T4, whereas D2 is deactivated by T4 and activated in TH deficiency (21). Thus, in a high serum T4/T3 serum ratio, TH signaling is reduced in D2-expressing tissues, whereas D1 is activated, catalyzing the T4-to-T3 conversion greatly contributing to the elevated serum levels of T3 (13). There is a third enzyme, type III iodothyronine selenodeiodinase (D3), which catalyzes the T4-to-reverse T3 (rT3) and T3-to-T2 conversion reactions. Considering that rT3 and T2 are inactive metabolites, D3 terminates TH action (13). D3 is expressed in the skeleton during prenatal development, but not during postnatal development and in mature skeleton (22).

The C3H/HeJ (C3H) inbred mouse has an inherited D1-deficiency. The mRNA expression and activity of D1 in the liver and kidney are only 5–10% of that in the C57BL/6J (B6) inbred strain, which presents higher D1 mRNA expression (23, 24). Low activity cosegregated with a series of five GCT repeats located in the 5′-flanking region of the C3H dio1 (D1 mouse gene) that impairs C3H promoter potency and provides a partial explanation for the lower D1 expression and activity (25). Despite marked D1 deficiency, the decrease in T3 production is compensated by a decrease in T4 clearance, resulting in increased serum T4, normal serum T3, and a gross euthyroid phenotype with normal bone growth (23), which is also observed in mice with global gene deletion of D1 (26), and is consistent with the lack of D1 activity in the skeleton (18, 27).

It is noteworthy that C3H and B6 mice, in addition to the D1 differences, present large differences in peak bone mass (28). C3H was described as having a phenotype of high bone mass because their peak bone density was found to be as much as 50 and 9% higher in the femur and vertebrae, respectively, than that of B6, which was characterized as presenting a phenotype of low bone mass (28). The genetic and physiological determination for differences in bone mass between these two inbred mouse strains is poorly understood and is likely to involve diverse factors. There is evidence that C3H mice have fewer osteoclasts (29), and that C3H mice presents higher intestinal calcium absorption associated with higher levels of 1,25 dihydroxivitamin D (28) and higher circulating levels of growth hormone and insulin-like growth factor-I than B6 mice (30).

To further characterize this mouse model (C3H) and to investigate if a global D1 deficiency could interfere in the effects of TH on bone, we evaluated bone parameters of C3H and B6 mice in situations of TH excess and deficiency. We found that abnormal TH status differentially affects bone mass accrual and bone strength, but not bone growth in C3H vs. B6 mice. In addition, this study provides evidence that both D1 deficiency and the distinct genetic background are likely to contribute to the different skeletal responses of C3H and B6 mice to TH.

## Materials and Methods

### Animals and Treatment

All experimental procedures were performed in accordance with the guidelines of the Standing Committee on Animal Research of the University of Sao Paulo. Female C3H and B6 mice were purchased from the Isogenic Mice Facility of the Department of Immunology, Institute of Biomedical Sciences, University of Sao Paulo and maintained under controlled conditions of light and temperature (12/12 h dark/light cycle at 25°C). All animals were kept in plastic cages, five to six per cage, and had free access to food (rat chow containing 1.4% Pi, 0.7% Ca, and 4.5 IU/g of vitamin D) and water. In a first set of experiments, 4-week old mice of both strains were randomly divided (*n* = 5 per group) into (i) euthyroid (Eut), untreated animals; (ii) hypothyroid (Hypo), induced by adding metimazole (MM; 0.1%) and NaClO${}_{4}^{-}$ (P; 1%) to the drinking water; (iii) 1xT4, animals treated with MM+P receiving 1 μg/100 g BW of T4 per day ip, which is equivalent to 1 physiological dose of T4 (31); and (iv) 10xT4, animals treated with MM+P receiving 10 μg/100 g BW of T4 per day ip, which is equivalent to 10 physiological doses of T4 (31). Treatment with MM+P and T4 started on the same day. All animals were killed by cervical dislocation after 16 weeks of treatment and the bones were processed for analysis. For determination of deiodinase activity and expression, 27–30-day-old C3H and B6 mice were randomly divided (n = 5 per group) into (i) Eut; (ii) Hypo, induced as described above; and (iii) Hyper, animals receiving 10xT4 per day (ip). After 8 days of treatment, the animals were killed by cervical dislocation and the tissues processed.

### Serum Concentration of T3, T4, and TSH and Bone Concentration of T3 and T4

Immediately before euthanizing the animals, the blood was collected from the retro-orbital plexus under anesthesia with CO_2_. Serum was separated by centrifugation and immediately frozen. Total T4 and T3 serum levels were measured by commercial RIA kits (RIA-gnost T4 and RIA-gnost T3, CIS bio international, France). For the T4 and T3 assays, standard curves were generated using charcoal-stripped mouse serum. Total T4 and T3 in the bone were determined accordingly to Obregón et al. (32) with modifications. Briefly, the lumbar vertebrae and both femurs, tibias, fibulas, scapulae, humerus, radius, and ulnas of each animal were pooled together and crushed in liquid nitrogen using a steel mortar and pestle set (Fisher Scientific International, Inc, Hampton, NH) until the bones became powder. The bone powder was transferred to pre-weighed microfuge tubes. Methanol, with 10^−3^M propylthiouracil (PTU) to avoid artifactual deiodination, was added in a volume that doubled the weight of the bone powder (2:1). The sample was vortexed for 2 min, kept on ice for 2 h and centrifuged (30 min 2,000 rpm, 4°C). The supernatant was transferred to a fresh tube (tube A) and the same volume of methanol with PTU (2:1) was added to the pellet. The sample was revortexed for 2 min, kept on ice for 15 min and recentrifuged (30 min, 2,000 rpm, 4°C). The supernantant was transferred to tube A and dried in a Speedvac® at 30°C (Eppendorf, Hamburg, Germany). one hundred fifty microliter of pre-tested mouse striped serum was added to tube A. The sample was vortexed for 1 min and total T4 and T3 levels were measured by RIA as described above. The values obtained were normalized by the weight of the bone powder and the results were expressed in nanograms per gram of bone. TSH levels were measured by Elisa assay (Rocky Mountain Diagnostics; Rat kit) following the manufacturer's instructions. The obtained values were expressed in picograms per milliliter.

### D1 and D2 Activity

After euthanasia, both femurs and the liver of each animal were dissected and cooled with liquid nitrogen. The bones were freed from bone marrow, homogenized as previously described (18) and protein concentration was quantified. The liver samples were homogenized on ice in buffer containing 1 x PE (0·1 M potassium phosphate and 1 mM EDTA), 0.25 M sucrose and 10 mM dithiothreitol (DTT) (pH 6.9), and then, the protein concentration was quantified. Protein concentration was quantitated by Bradford assay using BSA as a standard. Deiodination activity was assayed in the extracts of bone tissue and liver as previously described (22). Briefly, D1 or D2 assays were performed using 100–300 μg tissue protein, 1 μM or 1 nM unlabeled T_4_ in a total volume of 300 μl PE buffer containing 20 mM DTT, and approximately 100.000 cpm [^125^I] T_4_ (Amersham Biosciences, Piscataway, NJ, USA). Incubations were carried out at 37°C for 60–120 min. The reaction was terminated by adding 200 μl of horse serum and 100 μl of 50% trichloroacetic acid (TCA).

### RNA Isolation and PCR Analysis

The femurs of C3H and B6 mice were dissected, processed as previously described (22), and the total RNA was then extracted using Trizol (Invitrogen, Calbard, CA) following the manufacturer's instructions. Total RNA was also isolated from 50 to 100 mg of the liver using Trizol. The RNA concentration of each sample was quantified by spectrophotometry and the RNA was then visualized in agarose gels to ensure integrity. The mRNA expression of monocarboxylate transporter 8 (MCT8) and L-type amino acid transporters 1 and 2 (LAT1 and LAT2) and the thyroid hormone receptor alpha (TRα) and TRβ1 were analyzed by real-time PCR as previously described (33) using the following sets of primers: MCT8 (NM_009197.2) (F:CCCTGGACTTAAGAAGATATACTTGCA; R:CCCGAAGTCCCGGCATA), LAT1 (NM_011404.3) (F:CTACTTCTTTGGTGTCTGGTGGAA; R:GAGGTACCACCTGCATCAACTTC), LAT2 (NM_016972.2) (F:GACATCGGCTCGTTGCT; R:TGTAAGGATCCACAAGTCCTCAGT), TRα1 (NM_178060) (F: GCTGTGCTGCTAATGTCAACAGA; R: GCCTCCTGACTCTTCTCGATCTT),; TRβ1 (NM_001113417) (F:AAGCCACAGGGTACCACTATCG;R: GGAGACTTTTCTGAATGGTTCTTCTAA). Semiquantitative RT-PCR technique was used to determine the expression of D1 transcripts in the RNA samples of the liver as previously described (34), using the specific oligonucleotides derived from the coding region of the mouse D1 (sense: 5′ GCC ACT TCT GCC CCG TGC TGAG 3′ and antisense: 5′ CTG CCT TGA ATG AAATCC CAG ATG T 3′).

### Bone Densitometry

Bone mineral density (BMD) was measured by dual-energy X-ray absorptiometry (DXA)^1^ using the pDEXA Saber Bone Densitometer and the pDEXA Saber Software version 3.9.4 (Norland Medical Systems, Inc, Fort Atkinson, WI, U.S.A.), both specially designed for small animals. The animals were anesthetized with a ketamine-xylazine cocktail (10 and 30 mg/kg BW) and scanned in the prone position. The animals were subject to a basal scan and to a scan every 4 weeks of treatment. The bone regions analyzed were the total body, excluding the head and the tail; the lumbar vertebrae (L1-L6); and both femurs and tibias. The femoral and tibial BMDs were expressed as the mean of the left and right limbs for each animal. The precision *in vivo* was evaluated by calculating the coefficient of variation (CV = 100xSD/mean) of six repeated measurements of 2-month-old female mice. The animal was repositioned after each scan. The CV of BMD for total body, lumbar spine, femur and tibia were 0.6, 1.7, 0.4, and 0.3%, respectively. The precision *in vitro* was also expressed as CV and calculated by measuring the BMD of a phantom, with a nominal density of 0.929 g/cm^2^. This CV was 0.8% throughout the experiment.

### Body, Tibia, and Femur Longitudinal Growth

The length of the femur and tibia was measured indirectly by DXA using the ruler tool provided by the pDEXA Saber Software. The length of the femur and tibia were measured from the proximal to the distal epiphysis. Just after the DXA scan and under anesthesia, the body length was measured directly with a ruler (Norland Medical Systems) from the tip of the snout to the base of the tail. The longitudinal growth of each segment was calculated by the difference of the basal to the final measurements (final length–basal length).

### Three-Point Bending Test

The right femurs were submitted to the three-point bending test in an Instron testing machine (Model 4444, Instron Corporation, MA, USA). All femurs were tested in the same orientation: the anterior cortex was placed in compression and the posterior cortex in tension during the test. Each femur was placed on two supports spaced 6 mm apart. The bending load was applied at the midpoint, by a round loading contact with a diameter of 3 mm at a constant displacement rate of 5 mm/min until the bone fractured. The fracture was taken as complete loss of load carrying ability. A small preload (5% of the average maximal load) was applied before actual testing to stabilize the specimen. Load-displacement data were collected by a computerized data acquisition system at a sampling rate of 80 Hz during the bending test. The maximum load, which is a measure of strength recorded during the test, was determined.

### Statistical Analysis

One-way analysis of variance (ANOVA) was used to compare more than two groups and was always followed by the Student-Newman-Keuls test to detect differences between groups. Student-*t* test was used to compare two groups. For all tests, *p* < 0.05 was considered statistically significant. All results are expressed as the mean ± standard error of the mean (SEM). For statistical analysis, we used the GraphPad Instat Software (GraphPad Software Inc., San Diego, CA, USA).

## Results

### Effect of Thyroid Hormone Deficiency and Excess on BMD, Body Weight, and Body and Bone Growth

**Figure 1:**
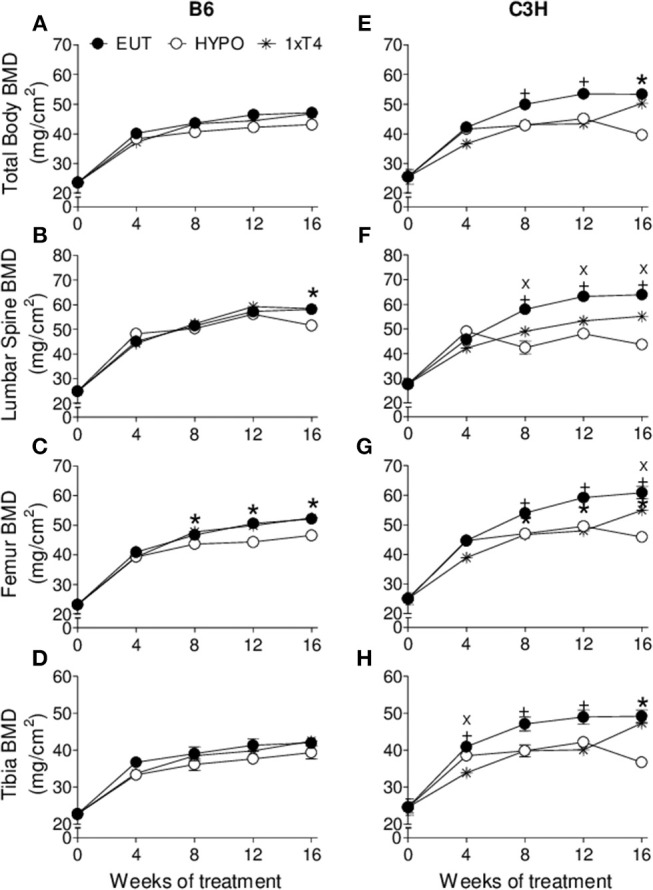
Effect of thyroid hormone deficiency on BMD. **(A,E)** total body, **(B,F)** lumbar spine, **(C,G)** femur, and **(D,H)** tibia of **(A–D)** B6 and **(E–H)** C3H mice. Euthryoid (Eut); Hypothyroidism (Hypo), induced by metimazole and NaClO${}_{4}^{-}$ (MM+P); MM+P-treated mice receiving 1 μg/100 g BW of T4 per day ip (1xT4). Each point is the mean ± SEM (n = 5/group). **+** *p* < 0.001, Eut vs. Hypo and 1xT4; **X** *p* < 0.001, Hypo vs. T4; ^*^p < 0.001, Hypo vs. Eut and T4 by Student-Knewman-Keuls test.

**Figure 2:**
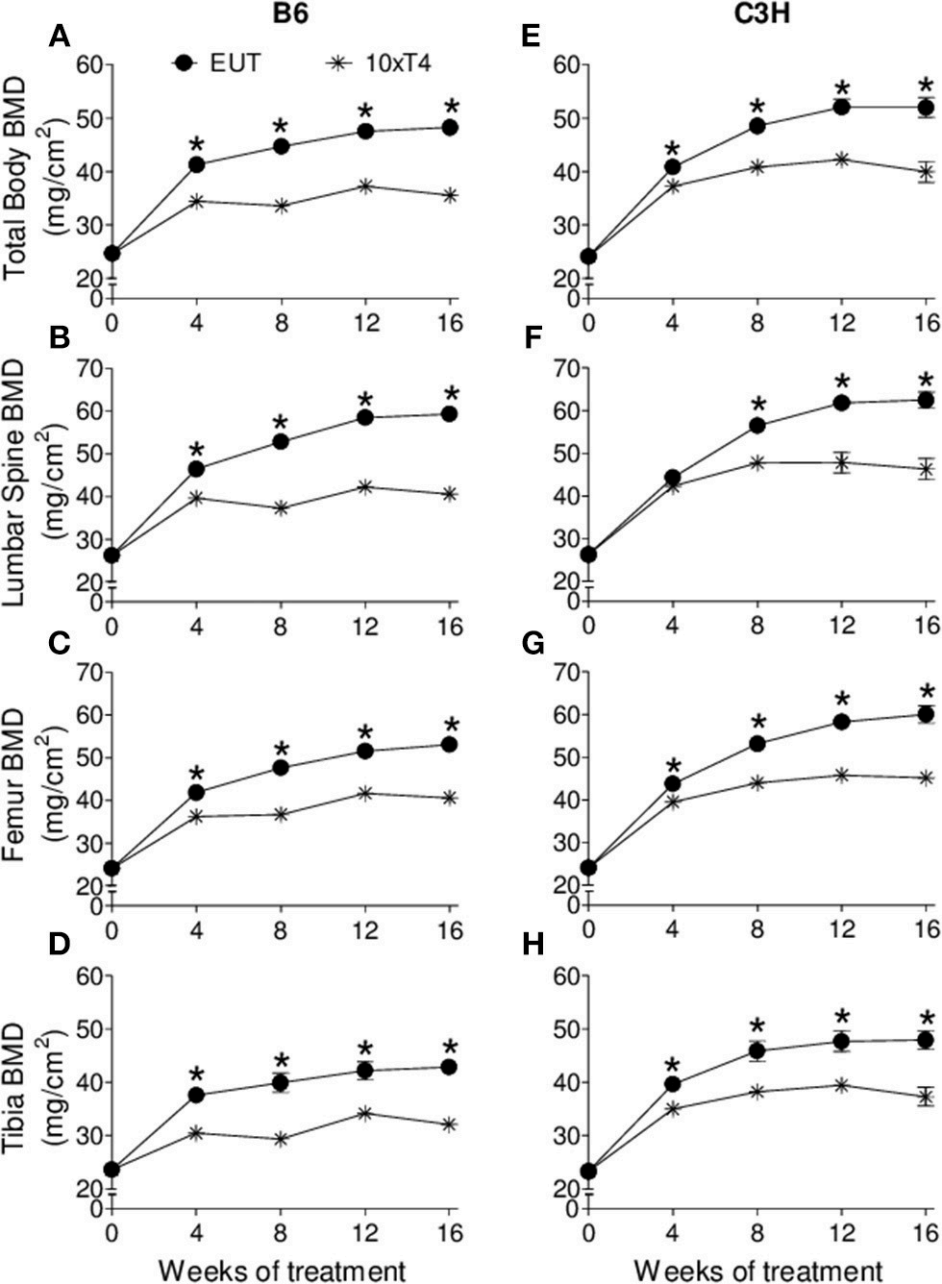
Effect of thyroid hormone excess on BMD. **(A,E)** total body, **(B,F)** lumbar spine, **(C,G)** femur, and **(D,H)** tibia of **(A–D)** B6 and **(E–H)** C3H mice. Euthryoid (Eut); Metimazole- and NaClO${}_{4}^{-}$-treated mice receiving 10 μg/100 g BW of T4 per day ip (10xT4). Each point is the mean ± SEM (*n* = 5/group). ^*^p < 0.001, Eut vs. 10xT4 by Student-*t* test.

**Figure 3:**
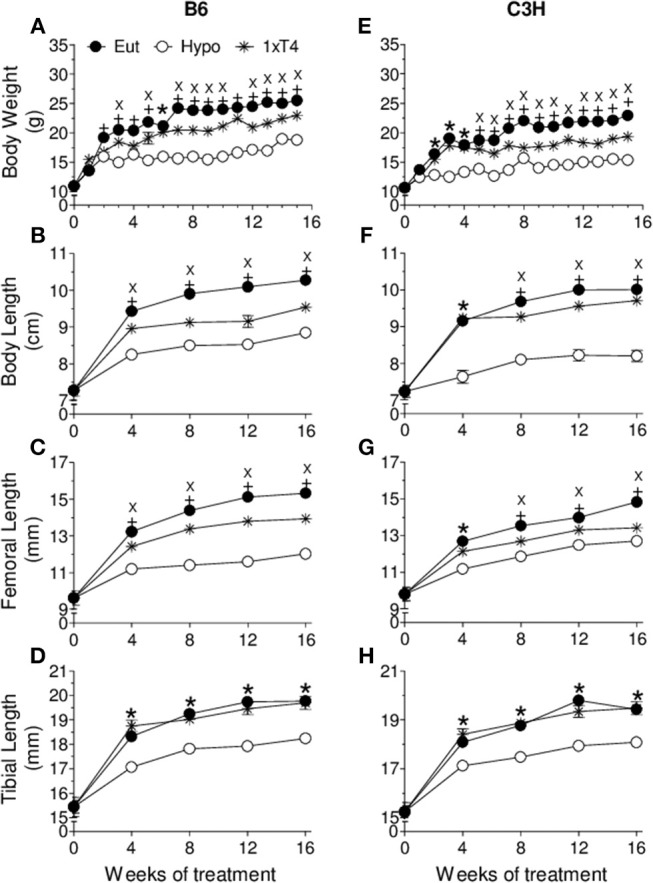
Effect of thyroid hormone deficiency on anthropometric parameters. **(A,E)** body weight, **(B,F)** body length, **(C,G)** femoral length, and **(D,H)** tibial length of **(A–D)** B6 and **(E–H)** C3H mice. Euthryoid (Eut); Hypothyroidism (Hypo), induced by metimazole and NaClO${}_{4}^{-}$ (MM+P); MM+P-treated mice receiving 1 μg/100 g BW of T4 per day ip (1xT4). Each point is the mean ± SEM (*n* = 5/group). **+** *p* < 0.001, Eut vs. Hypo and 1xT4; **X** *p* < 0.001, Hypo vs. T4; ^*^p < 0.001, Hypo vs. Eut and T4 by Student-Knewman-Keuls test.

**Figure 4:**
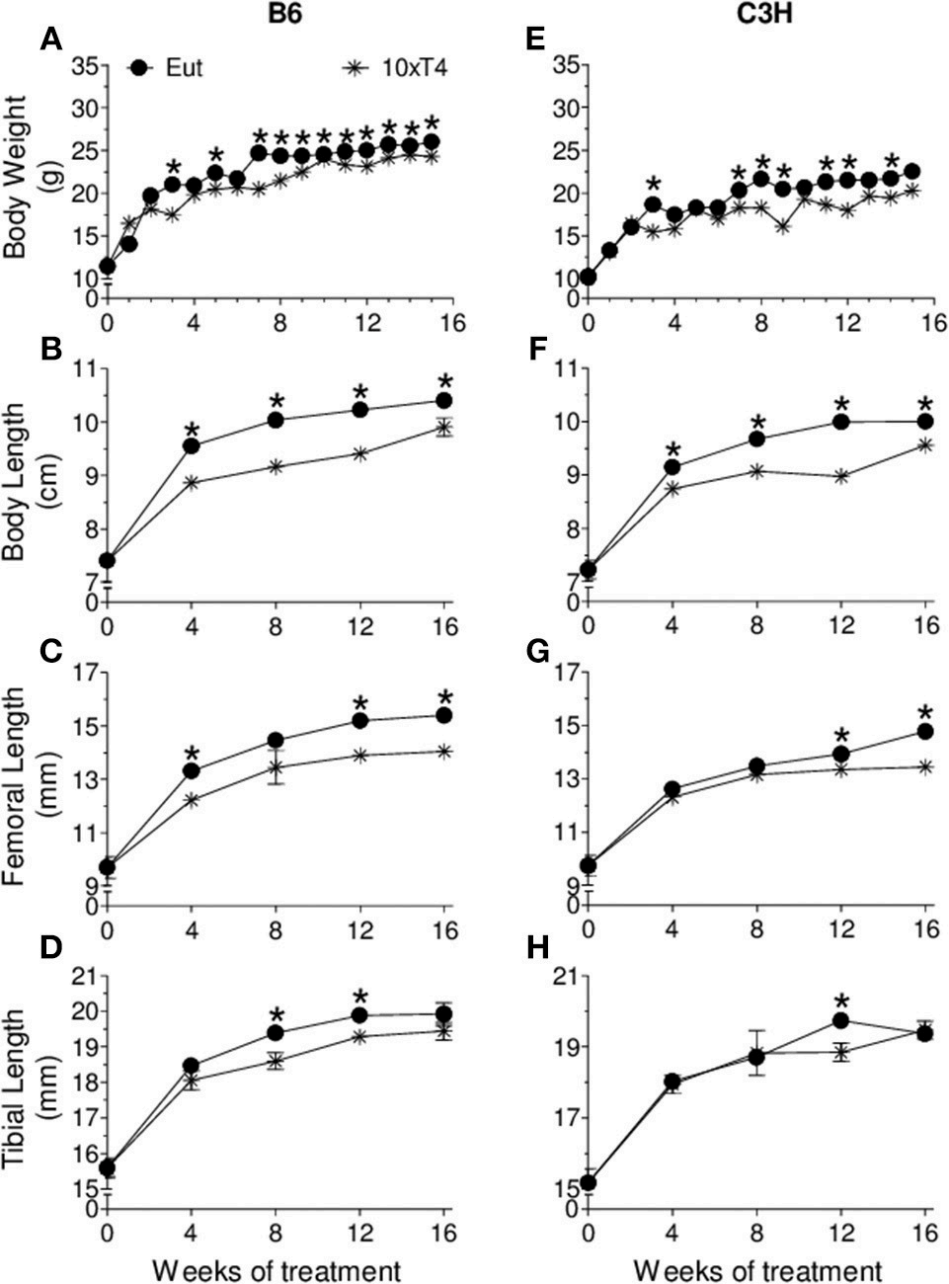
Effect of thyroid hormone excess on anthropometric parameter. **(A,E)** body weight, **(B,F)** body length, **(C,G)** femoral length, and **(D,H)** tibial length of **(A–D)** B6 and **(E–H)** C3H mice. Euthryoid (Eut); Metimazole- and NaClO${}_{4}^{-}$-treated mice receiving 10 μg/100 g BW of T4 per day ip (10xT4). Each point is the mean ± SEM (n = 5/group). ^*^p < 0.001, Eut vs. 10xT4 by Student-*t* test.

At the basal state, when animals were 4 weeks old, there were no differences in body weight, body length, femoral length and tibial length between C3H and B6 mice. However, as expected, BMD of C3H mice was higher than that of B6 mice in the total body (9%, *p* = 0.04), lumbar spine (12%, *p* = 0.02) and femur (9%, *p* = 0.04), which confirms the typical bone mass differences between these two strains of mice. Interestingly, BMD was shown to respond differently to thyroid status in C3H vs. B6 mice (Figures 1, 2). Hypothyroidism only slightly limited the gain of BMD in the lumbar spine and femur of B6 mice (Figures 1B,C), but significantly impaired BMD acquisition in all skeletal sites of C3H mice (Figures 1E–H). In addition, 1xT4 only partially rescued BMD gain in C3H mice (Figures 1E–H), while it completely rescued the gain of BMD in the femur and vertebrae of B6 mice (Figures 1B,C). It is remarkable that TH excess induced by 10xT4-treatment, significantly impaired BMD gain in all skeletal sites of both strains (Figure 2). However, it was slightly more severe in B6 mice than in C3H mice. As shown in Table 1, the percentual gain of total body and tibia BMD in 10xT4-treated animals was significantly lower in B6 mice than in C3H mice, while there were no differences between strains in Eut animals. On the other hand, TH deficiency and excess limited the gain of body weight, body length, femoral length and tibial length in both strains of mice in a similar fashion (Figures 3, 4). In C3H and B6 animals, 1xT4 totally rescued the growth deficit induced by hypothyroidism in the tibia (Figures 3D,H) and partially rescued the deficits observed in body weight, body length and femoral length, where we could see that 1xT4-treatment resulted in intermediate values of these parameters between Eut and Hypo groups.

**Table 1:** Percentual Gain of BMD in C3H and B6 Mice.

**ΔBMD %**	**Eut**	**10xT4**	***P* (*t*-test)**
**TOTAL BODY**			
B6	105.5 ± 6.3	41.7 ± 2.9	< 0.0001
C3H	123.2 ± 13.1	68.78 ± 10.9	0.0104
*P* (t-test)	0.25	0.03	
**LUMBAR SPINE**			
B6	146.79 ± 12.9	51.98 ± 5.1	< 0.0001
C3H	146.61 ± 16.9	81.72 ± 12.9	0.0126
*P* (*t*-test)	0.99	0.06	
**FEMUR**			
B6	133.18 ± 9.7	65.08 ± 3.3	< 0.0001
C3H	155.19 ± 12.4	92.67 ± 12.5	0.0066
*P* (*t*-test)	0.19	0.06	
**TIBIA**			
B6	90.62 ± 6.8	34.15 ± 2.1	< 0.0001
C3H	114.95 ± 10.1	62.49 ± 11.3	0.0080
*P* (*t*-test)	0.07	0.03	

*Values are the mean ± SEM (n = 5 per group).The percentual gain of BMD was calculated from the basal BMD to the final BMD as follows: ΔBMD% = (_final_BMD–_basal_BMD) x 100/_basal_BMD. Significance between groups was determined by Student t-test*.

### Effect of Thyroid Hormone Deficiency and Excess on Femoral Maximum Load

**Figure 5:**
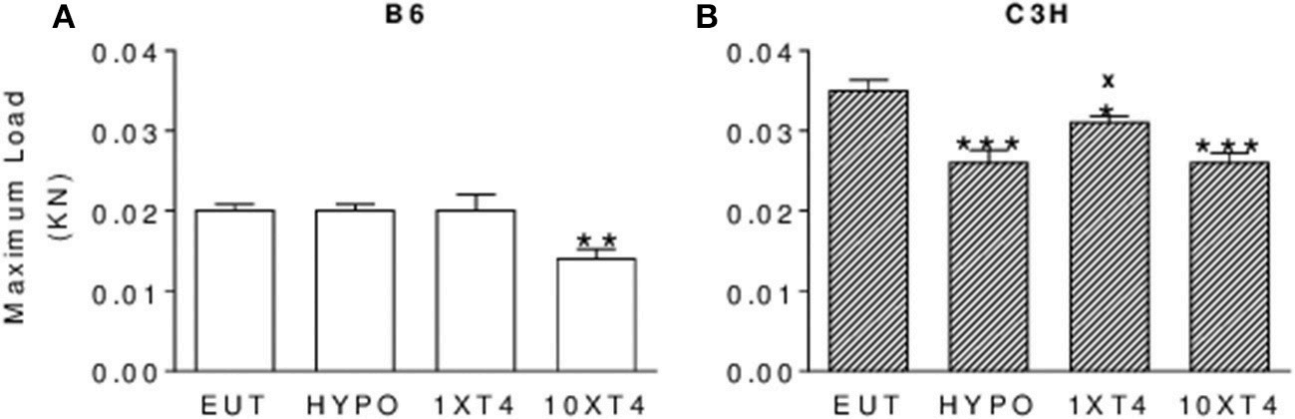
Effect of thyroid hormone deficiency and excess on bone strength. Bone strength was evaluated by measuring the maximum load (KN) applied to the femur of **(A)** B6 and **(B)** C3H mice by the three-point bending test. Euthryoid (Eut); Hypothyroid (Hypo), induced by metimazole and NaClO${}_{4}^{-}$ (MM+P); MM+P-treated mice receiving 1 or 10 μg/100 g BW of T4 per day ip (1xT4 and 10xT4, respectively). Bars represent the mean ± SEM (n = 5/group). ^*^p < 0.05, ^**^p < 0.01, ^***^p < 0.001 vs. Eut; **x** *p* < 0.05 vs. Hypo; **+** *p* < 0.05 vs. 1xT4 by Student-Knewman-Keuls test.

In agreement with the effects on BMD, TH deficiency also differently affected the maximum load in the femur of C3H and B6 mice. Hypothyroidism did not change the maximum load in B6 mice (Figure 5A), but significantly decreased this biomechanical parameter in C3H mice, which was only partially rescued by 1xT4 (Figure 5B). On the other hand, TH excess induced by 10xT4 treatment significantly decreased this parameter in both strains (Figures 5A,B).

### Serum Levels of T3, T4, and TSH and Bone Tissue Levels of T3 and T4

As expected, there were no differences in serum levels of T3 between Eut animals of both strains, while serum levels of T4 were elevated in C3H as compared with B6 mice (Table 2), which is considered a mechanism to compensate for D1 deficiency in C3H mice (23). It is noteworthy that in 10xT4-treated animals serum levels of T3 were higher (*p* < 0.0278) while serum levels of T4 were lower (*p* = 0.0019) in B6 mice than in C3H mice. No differences in serum levels of T3 and T4 between strains in Hypo and 1xT4-treated mice were observed. Among groups of the same lineage, characteristic differences in serum levels of TH were observed (Table 2). Both B6 and C3H mice from Hypo groups presented lower serum levels of T3 and T4 as compared with their respective Eut groups. Accordingly, B6 and C3H mice treated with 10xT4 presented higher serum levels of T3 and T4 as compared with Eut animals. As anticipated, both mice strains presented elevated and undetectable TSH serum levels in Hypo and Hyper states, respectively (Table 2). In addition, TSH serum levels were not different between C3H and B6 mice in euthyroidism and hypothyroidism. Interestingly, we found that bone levels of T3, but not T4, were significantly lower in Eut C3H vs. Eut B6 mice (27%, *p* < 0.04). On the other hand, there were no differences in bone levels of T3 and T4 between Hypo animals of both strains. As expected, bone levels of T4 and T3 were significantly reduced in Hypo vs. Eut animals of both strains (Table 3).

**Table 2:** Serum Parameters.

	**Eut**	**Hypo**	**1XT4**	**10XT4**	***P***
**T3 (ng/ml)**					
B6	0.43 ± 0.04	0.06 ± 0.03^a^	0.23 ± 0.03	0.99 ± 0.06^b^, ^d^, ^e^	<0.0001
C3H	0.46 ± 0.03	0.13 ± 0.01^b^	0.29 ± 0.13	0.81 ± 0.03^c^, ^d^, ^e^	<0.0001
*P* (*t*-test)	n.s.	n.s.	n.s.	0.0278	
**T4 (ng/ml)**					
B6	42.2 ± 3.14	6.80 ± 1.42^c^	34.8 ± 1.07^c^, ^d^	67.6 ± 3.6^c^, ^d^, ^e^	<0.0001
C3H	53.1 ± 3.16	6.40 ± 2.64^c^	38.5 ± 1.95^b^, ^d^	95.6 ± 5.0^c^, ^d^, ^e^	<0.0001
*P* (*t*-test)	0.04	n.s.	n.s.	0.0019	
**TSH (pg/ml)**					
B6	66.3 ± 4.23	620 ± 61.44^c^	n.m.	und.	<0.0001
C3H	84.1 ± 7.93	625.5 ± 18.47^c^	n.m.	und.	<0.0001
*P* (*t*-test)	n.s.	n.s.			

*Values are the mean ± SEM (n = 5 per group). Significance among groups was determined by ANOVA followed by Student-Newman-Keuls test or by Student t-test as specified; n.s. = non-significant; n.m. = not measured; und. = undetectable*.

a *p < 0.05 vs. Eut*.

b *p < 0.01 vs. Eut*.

c *p < 0.001 vs. Eut*.

d *p < 0.001 vs. Hypo*.

e *p < 0.001 vs. 1xT4*.

**Table 3:** T3 and T4 Concentration in the Skeleton.

	**Eut**	**Hypo**	***P* (*t*-test)**
**T3 (ng/g)**			
B6	0.47 ± 0.05	0.11 ± 0.04	0.0002
C3H	0.34 ± 0.03	0.21 ± 0.05	0.04
*P* (*t*-test)	0.04	n.s.	
**T4 (ng/g)**			
B6	1.12 ± 0.21	0.42 ± 0.11	0.04
C3H	1.28 ± 0.10	0.71 ± 0.19	0.03
*P* (*t*-test)	n.s.	n.s.	

*T3 and T4 concentration in the skeleton were measured by RIA in a pool of bones (lumbar vertebrae and both femurs, tibias, fibulas, scapulas, humerus, radius and ulnas) of each animal. Results are expressed in nanograms per gram of bone. Values are the mean ± SEM (n = 3–5 per group). Significance among groups was determined by Student t-test as specified; n.s. = non-significant*.

### Expression of Thyroid Hormone Transporters and Thyroid Hormone Receptors

**Figure 6:**
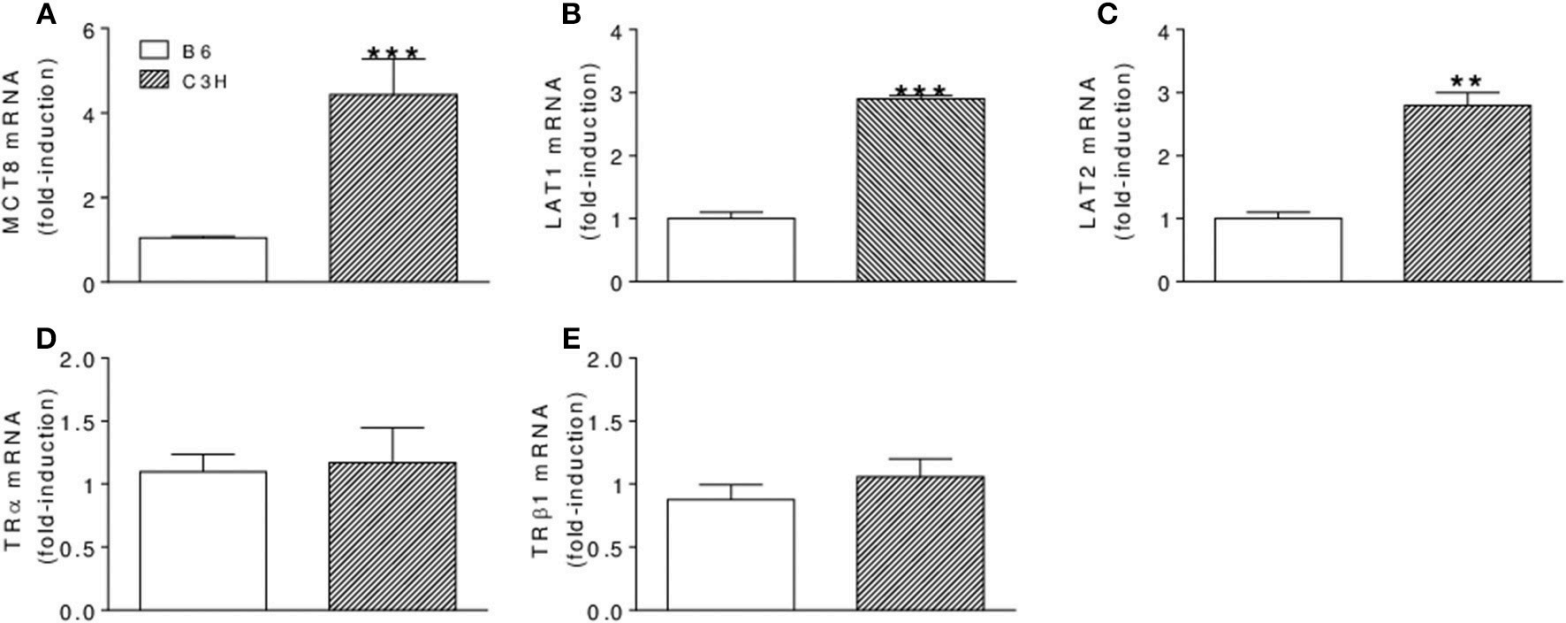
Femoral mRNA expression of thyroid hormone transporters **(A–C)** and thyroid hormone receptors **(D–F)** in B6 and C3H mice. **(A)** MCT8, **(B)** LAT1, **(C)** LAT2, **(D)** TRα, and **(E)** TRβ1 mRNA expressions were assessed by real-time PCR and normalized to β-actin mRNA expression. Bars represent mean ± SD (*n* = 5/group). ^**^p < 0.01 and ^***^p < 0,001 vs. B6 by Student Knewman-Keuls Test.

Considering that cellular influx/efflux of iodothyronines is likely to determine bone levels of TH, we next evaluated the femoral expression of three plasma membrane TH transporters that were shown (33) to be expressed in the skeleton of mice: monocarboxylate transporter 8 (MCT8) and L-type amino acid transporters 1 and 2 (LAT1 and LAT2, respectively). Interestingly, we found that the mRNA expression of these TH transporters was significantly increased in the femur of C3H vs. B6 mice (2.8 to 4.4 folds) (Figures 6A–C). We also compared the mRNA expression of TRα1 and TRβ1 in the femur of C3H vs. B6 mice. No differences were observed between the two mouse strains for these receptors in euthyroidism (Figures 6D,E), hypothyroidism and hyperthyroidism (data not shown).

### D1 and D2 mRNA Expression and Activity

**Figure 7:**
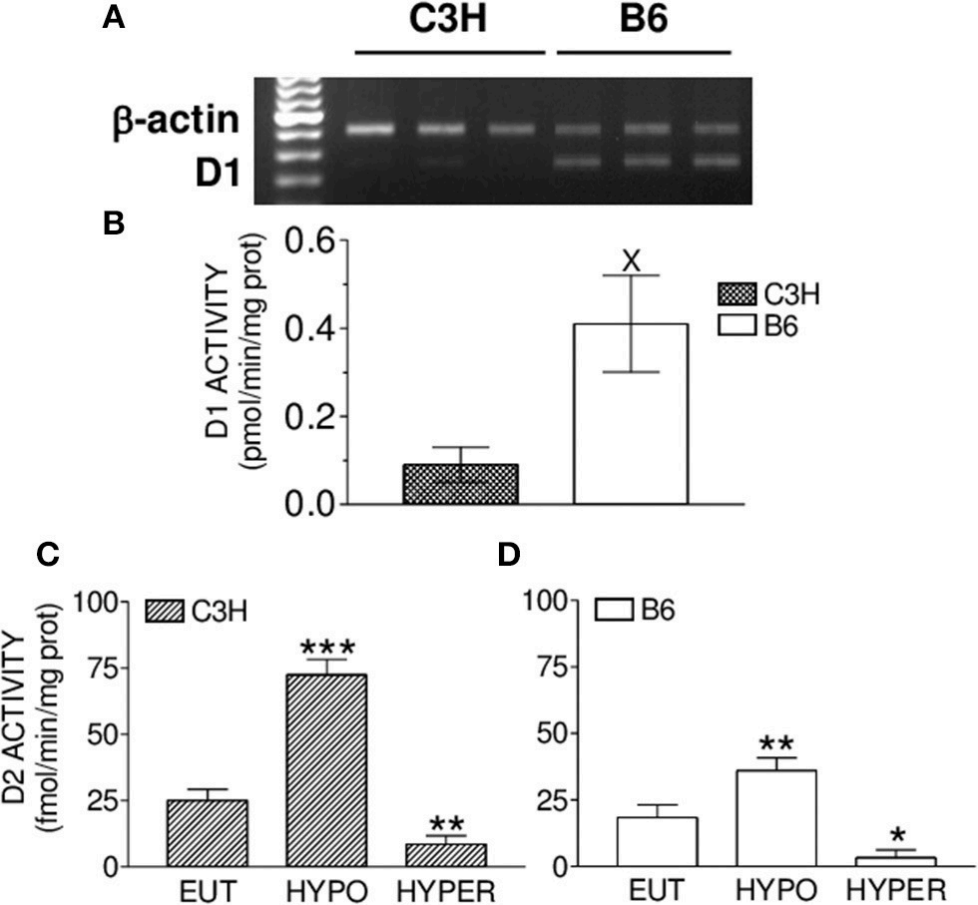
Liver D1 mRNA expression and activity and femoral D2 activity. **(A)** Semiquantitative RT-PCR analysis showing the levels of liver D1 and β-actin mRNA. **(B)** Liver D1 activity. Effect of hypothyroidism (HYPO) and hyperthyroidism (HYPER) on femoral D2 activity of **(C)** C3H and **(D)** B6 mice. Bars represent mean ± SEM (*n* = 5/group). ^*^p < 0.05, ^**^p < 0.01, and ^***^p < 0.001 vs. Eut by Student Knewman-Keuls Test; ^X^ *p* < 0.01 by Student *t*-test.

Considering that D2, but not D1, is present in the bone tissue of mice (18), we compared D2 activity in the femur of B6 and C3H mice to investigate if the differential skeletal responses of C3H and B6 mice could be explained by a diverse D2 activity between strains. We first confirmed that D1 mRNA and activity were significantly higher in the liver of B6 as compared with C3H mice (Figures 7A,B). As it is characteristic for D2, its activity was increased in HYPO animals and decreased in thyrotoxic animals (treated with 10xT4) of both strains (Figures 7C,D). It is interesting that femoral D2 activity was 50% higher in Hypo C3H mice as compared with Hypo B6 mice (72 ± 5.9 vs. 36 ± 4.9 fmol/min/mg prot, *p* < 0.01), while there were no differences in D2 activity between strains in Eut and Hyper animals.

## Discussion

We showed that TH deficiency and excess impaired body weight gain and the longitudinal growth of the body, femur and tibia in a similar fashion in C3H and B6 mice (Figures 3, 4). On the other hand, the response of bone mass accrual and bone strength to thyroid status showed marked differences between these two strains of inbred mice. While hypothyroidism only slightly impaired the gain of bone mass in the femur and lumbar vertebrae of B6 mice (Figure 1), it significantly impaired the gain of BMD in all skeletal sites evaluated of C3H mice (femur, tibia, lumbar spine and total body). In addition, a physiological dose of T4 (1xT4) only partially rescued BMD gain in C3H mice but totally rescued bone mass acquisition in the femur and vertebrae of B6 mice. The analysis of bone strength in the femur by the three-point bending test (expressed as maximum load) showed that hypothyroidism decreased bone strength only in C3H but not in B6 mice (Figure 5). It is interesting that thyrotoxicosis limited BMD gain and decreased bone strength in both strains but the effects on BMD were somewhat more severe in B6 mice (Table 1). Therefore, C3H animals showed to be much more susceptible to the deleterious effects of hypothyroidism on peak bone mass acquisition and bone strength than B6 mice. On the other hand, B6 mice showed to be more vulnerable to the effects of thyrotoxicosis on bone mass than C3H mice.

An explanation for the distinctive effects of TH on BMD and bone strength between strains may be based on the fact that C3H and B6 mice present basal differences on the bone remodeling rate. It was shown that C3H mice present low bone turnover, while B6 mice present a high bone remodeling rate (35). Since hypothyroidism leads to a decrease in bone remodeling (36), followed by a marked reduction in the number of osteoblasts and in the bone formation rate (8), it is possible that TH deficiency reduced bone formation and metabolism to a more critical level in C3H mice than in B6 mice. On the other hand, it is known that thyrotoxicosis increases the remodeling rate with an unbalance between bone formation and resorption, which leads to bone loss (3). Therefore, considering that B6 mice have a higher basal bone remodeling rate than C3H mice (35), it is also possible that TH excess increased bone turnover to a more crucial level in B6 than in C3H mice.

Another explanation for the different responses of bone mass and bone resistance to TH between strains could be a higher induction of thyrotoxicosis in B6 mice and a higher induction of hypothyroidism in C3H mice. In fact, we found higher serum levels of T3 and lower serum levels of T4 in 10xT4-treated B6 mice as compared with 10xT4-treated C3H mice (Table 2). It is well-known that D1 activity is increased by T4 (13). Therefore, the conversion of T4 to T3 was certainly increased by 10xT4-treatment in C3H and B6 mice, which is confirmed by the higher levels of T3 in 10xT4-treated mice of both strains in comparison with Eut animals (Table 2). However, the deficiency of D1 in C3H animals probably lead to a lower conversion of T4 to T3, which explains the higher levels of serum T4 and lower levels of serum T3 in C3H mice vs. B6 mice when treated with 10xT4. These results suggest that global differences in D1 activity between these two strains of mice explain, at least partially, the TH excess-induced differences in serum levels of T3 and T4 and in BMD between strains. Thus, D1 seems to assume an indirect but relevant role to bone mass when the systemic homeostasis is challenged by TH excess, regardless of its irrelevance to the skeleton under euthyroid conditions (37).

On the other hand, a higher induction of systemic hypothyroidism in C3H vs. B6 mice was not confirmed since there were no differences in serum levels of TH between hypothyroid animals of both strains (Table 2). We, therefore, investigated if local (bone) hypothyroidism was more intense in C3H vs. B6 mice, which was not confirmed as well. Nevertheless, it was remarkable to note that bone levels of T3 were significantly lower in Eut C3H vs. Eut B6 mice (27 %, *p* < 0.04). Considering the role of TH in activating bone metabolism (36), it is possible that the low bone levels of T3 (local hypothyroidism) in Eut C3H contributes to the characteristic low bone metabolism observed by others in this mouse model (35). It is noteworthy that femoral mRNA expression of TRs was not different between C3H and B6 mice, suggesting that the lower bone levels of T3 were not compensated by upregulation of TRs.

To get insights if the lower concentrations of T3 in the bone of Eut C3H vs. Eut B6 mice could be related to a deficiency in TH transport across the plasma membrane, we evaluated the femoral expression of three TH transporters that were shown to be expressed in the skeleton of mice, MCT8, LAT1 and LAT2 (33). This appears not to be true, since the mRNA expression of these three TH transporters was significantly increased in the femur of C3H vs. B6 mice (Figure 6), which, in turn, may reflect a bone effort to increase local levels of TH. This finding is partially corroborated by a previous study, where we showed that MCT8 mRNA expression is upregulated by low levels of TH in the skeleton of mice and in osteoblast-like cells (33).

Considering that C3H mice are D1 deficient (25) and that D2, but not D1, is widely expressed in the skeleton of mice (18, 19), we decided to investigate if differences in bone D2 activity could explain the lower bone levels of T3 in Eut C3H vs. Eut B6 mice and/or could play a role in the diverse skeletal responses between both strains of mice. We first confirmed that C3H mice presented low mRNA levels and activity of D1 in the liver (Figures 7A,B). Next, we showed that hypothyroidism increased while TH excess decreased D2 activity in the femur of C3H and B6 mice (Figures 7C,D), as it is characteristic for this enzyme (13). In addition, we found that there were no differences in femoral D2 activity between strains in both Eut and 10xT4-treated mice. This was somewhat unpredictable for the Eut group based on previous studies that have shown that D2 activity is about 50% lower in the brain and pituitary of C3H animals as compared with B6 mice, probably due to the high levels of serum T4 (23). Nevertheless, in agreement with our findings, studies have shown that D2 activity in brown adipose tissue is also unaffected by higher serum levels of T4 in C3H animals (34). It is noteworthy, however, that hypothyroidism increased femoral D2 activity 2-fold more in C3H vs. B6 mice, which probably explains why local levels of T3 were similar in HYPO animals of both strains in spite of being significantly lower in Eut C3H vs. Eut B6. The reason for this higher response of D2 to hypothyroidism in C3H mice is not clear since both bone concentration and serum levels of T3 and T4 were similar in hypothyroid animals of both strains. Altogether, these findings suggest that the regulation of bone D2 activity also depends on other factors than local concentration and serum levels of TH. In fact, there is evidence that growth factors, adrenergic signaling, environmental, and nutritional factors may influence deiodinase activities (13), although these enzymes are mainly regulated by thyroid hormones. Whereas, D1 activity is almost exclusively regultated by TH at the transcriptional level (38), D2 regulation by TH is much more complex, involving transcriptional, posttranscriptional and posttranslation mechanisms. Therefore, further studies are necessary to better understand how D2 activity is regulated in the bone of mice and to reveal the mechanism whereby D2 activity is higher in C3H vs. B6 mice under hypothyroid conditions. It is noteworthy that the higher increase in D2 activity in response to hypothyroidism in C3H vs. B6 mice probably contributed to the equal bone levels of T3 in the hypothyroid condition between the two mouse strains. Thus, D2 activity was not a factor that contributed to the skeletal differences between C3H and B6 mice in hypothyroid conditions. Nevertheless, the findings of the present study reinforce the idea that D2 has a role in the maintenance of TH levels in the bone.

Considering that bone levels of TH and TR were not different between hypothyroid animals (MM+P-treated mice) of both strains, the different genetic background of C3H and B6 mice should contribute to the diverse effects of hypothyroidism on bone mass and function between strains. In fact, a series of studies has shown that genetic factors influence bone mass acquisition or loss induced by non-genetic factors. Bouxsein et al. (39) showed that estrogen deficiency induced vertebral trabecular bone loss in four strains of mice, including B6, but not in C3H. In contrast, at the proximal tibia, C3H had a greater decline in trabecular bone volume than B6. It was also shown that bone mass of C3H mice is practically unaffected by disuse, while bone mass of B6 mice is significantly reduced by immobilization (35, 40). Nevertheless, the lower bone concentration of T3 in Eut C3H vs .Eut B6 mice may, indeed, be an important factor to the characteristic lower bone turnover presented by C3H animals, which in turn is likely to contribute to the higher responsiveness of bone mass and strength to the deleterious effects of hypothyroidism.

In summary, we found that C3H mice present a lower concentration of T3 in the skeleton than B6 mice, which may contribute the previously observed lower bone turnover rate in C3H compared to B6 strain (35). Another interesting finding was that the skeletons of C3H and B6 mice respond differently to TH excess and deficiency. We showed that high doses of T4 resulted in less elevated serum levels of T3 and were less deleterious to the bone mass acquisition of C3H than B6 mice, which is probably explained by the lower D1 activity in C3H mice. On the other hand, we showed that hypothyroidism barely impairs peak bone mass acquisition in B6 mice, but severely affects bone mass accrual in C3H mice, while there were no differences in serum and bone levels of T3 and T4 between hypothyroid animals of both strains. It suggests that the distinct effect of thyroid hormone deficiency between C3H and B6 mice depends on deiodinase-unrelated factors.

In conclusion, our findings show that C3H mice present lower bone levels of T3, which may contribute to the characteristic low bone turnover rate of this mouse model. In addition, our findings suggest that D1 deficiency contributes to the TH excess-induced differences in peak bone mass accrual between C3H and B6 mice, indicating that D1 assumes an indirect but relevant role to bone mass when the systemic homeostasis is challenged by TH excess, in spite of being irrelevant to the skeleton under euthyroid conditions. Our findings also suggest that other genetic factors, unrelated to deiodinases, but probably related to osteoblast and osteoclast activities, distribution, and physiology (41–44), and related to specific signaling pathways in these cells, account for the diverse skeleton responses to hypothyroidism between these two inbred mouse strains. Therefore, further studies are necessary to identify these genetic factors. Altogether, these findings highlight the caution against generalizing the skeletal effects of TH across different mouse strains and individuals.

## Ethics Statement

This study was carried out in accordance with the recommendations of the Standing Committee on Animal Research guideline. The protocol was approved by the Standing Committee on Animal Research of the University of Sao Paulo.

## Author Contributions

CZ: conceived and performed experiments, carried out data collection and analysis, and wrote the manuscript. TF, LC, FF, EB, JD, CW, and MM-R: performed experiments, carried out data collection and analysis, and reviewed the manuscript. KN and AM: conceived the study and experiments, carried out data analysis and reviewed the manuscript. CG: conceived the study and experiments, carried out data analysis and wrote the manuscript.

### Conflict of Interest Statement

The authors declare that the research was conducted in the absence of any commercial or financial relationships that could be construed as a potential conflict of interest.

## Footnotes

^1^*The abbreviation “DXA” was proposed by the International Society for Clinical Densitometry (ISCD) in 2003, as an alternative to “DEXA”. DXA has been preferentially used in the scientific articles*.
